# Immune Escape Mechanisms and Future Prospects for Immunotherapy in Neuroblastoma

**DOI:** 10.1155/2018/1812535

**Published:** 2018-02-25

**Authors:** Thitinee Vanichapol, Somchai Chutipongtanate, Usanarat Anurathapan, Suradej Hongeng

**Affiliations:** ^1^Division of Hematology and Oncology, Department of Pediatrics, Faculty of Medicine, Ramathibodi Hospital, Mahidol University, Ratchathewi, Bangkok 10400, Thailand; ^2^Pediatric Translational Research Unit, Department of Pediatrics, Faculty of Medicine, Ramathibodi Hospital, Mahidol University, Ratchathewi, Bangkok 10400, Thailand; ^3^Department of Cancer Biology, University of Cincinnati College of Medicine, Cincinnati, OH 45267, USA

## Abstract

Neuroblastoma (NB) is the most common extracranial solid tumor in childhood with 5-year survival rate of 40% in high-risk patients despite intensive therapies. Recently, adoptive cell therapy, particularly chimeric antigen receptor (CAR) T cell therapy, represents a revolutionary treatment for hematological malignancies. However, there are challenges for this therapeutic strategy with solid tumors, as a result of the immunosuppressive nature of the tumor microenvironment (TME). Cancer cells have evolved multiple mechanisms to escape immune recognition or to modulate immune cell function. Several subtypes of immune cells that infiltrate tumors can foster tumor development, harbor immunosuppressive activity, and decrease an efficacy of adoptive cell therapies. Therefore, an understanding of the dual role of the immune system under the influences of the TME has been crucial for the development of effective therapeutic strategies against solid cancers. This review aims to depict key immune players and cellular pathways involved in the dynamic interplay between the TME and the immune system and also to address challenges and prospective development of adoptive T cell transfer for neuroblastoma.

## 1. Introduction

Neuroblastoma (NB) is the most common extracranial solid tumor of early childhood, accounting for about 6% of all childhood cancers, with an incidence of 1/70,000 in children younger than 15 years [1]. It is a neuroblastic tumor arising from deregulation of the signaling pathways governing primitive sympathetic ganglion cell development that also include ganglioneuroblastoma and ganglioneuroma [2]. NB patients are subdivided into low-, intermediate-, and high-risk groups based on clinical stage, age at diagnosis, tumor histology, MYCN oncogene amplification, histology, and chromosomal ploidy. High-risk NB has a high recurrence rate. The most common sites for metastasis are bone marrow (BM), bone, lymph nodes, and liver [2]. The 5-year survival rate of high-risk patients remains around 40%, even after the use of multimodal intensive treatment [3]. Current standard therapy for high-risk patients includes induction chemotherapy and surgery, high-dose chemotherapy and radiation therapy with stem cell rescue, and anti-disialoganglioside (GD2) mAb ch14.18 combined with interleukin- (IL-) 2 and Granulocyte-Macrophage Colony Stimulating Factor (GM-CSF) [4]. Heterogeneity in clinical presentation and prognosis is a hallmark of NB, which can be attributed to molecular differences, including MYCN amplification and 1p deletions or 11q deletions. The most malignant forms have amplification of the MYCN oncogene. Taken together, the development of new and more effective immunotherapies is a high priority.

A good example of the promising therapy in NB is GD2-targeted immunotherapy. GD2 is a ganglioside uniformly expressed by NB, glioma, melanoma, and sarcomas cells and serves as a target for monoclonal antibody-based therapeutic intervention [5]. The use of anti-GD2 mAb plus systemic cytokines IL-2 and GM-CSF and retinoic acid therapy in clinical trials has shown promising results in patients with high-risk NB [6]. Recently, genetic engineering of T lymphocytes to express anti-GD2 chimeric antigen receptor (CAR) has been developed and tested in clinical trials. This approach represents the novel therapeutic measures in the fight against high-risk NB. Despite the success stories of CAR T cells in hematological malignancies, the efficacy of CAR T cells in solid tumors, including NB, can be complicated by the complex tumor microenvironment (TME), which may lead to therapeutic resistance, thus posing a significant challenge to the success in immunotherapy [7].

The appreciation of the TME has started when Stephen Paget proposed the “seed and soil” hypothesis in 1889 to explain the metastatic behavior of tumor cells (the “seed”) to the preferential metastatic sites (the “soil”) [8, 9]. The nonrandom patterns of tumor metastasis are the result of interactions between metastatic tumor cells and their organ microenvironment. This fact highlighted the importance of a complex relationship between tumor cells with host factors and nonmalignant cells. Cancerous cells reside in a specialized niche made up of stromal support cells, soluble factors, the vascular system, extracellular matrix proteins, and infiltrating immune cells. Secretory cytokines and autocrine and paracrine factors from tumor cells have a significant influence on the host immune response in order to alter conditions essential for tumor survival, development, and progression [10].

The notion that immune cells can recognize and eradicate nascent transformed cells can be dated back to the late 1950s when Burnet and Thomas introduced the theory of “immunosurveillance” [11]. Nonetheless, research over the past few decades prompted us to extend our interpretation into a conceptual model known as “cancer immunoediting” [11]. We have learned that the theory of “immunosurveillance” is only a part of the story. New data provides strong support for the view that both innate and adaptive immunity play multifaceted roles in tumor eradication and shaping tumor immunogenicity [12].

Cancer immunoediting consists of three sequential phases: elimination, equilibrium, and escape. The “elimination phase” is the modern concept of immune surveillance. Both innate and adaptive immunity play a role in recognition and fighting against tumors before they become clinically visible. The main effectors are CD8^+^ T cells, which recognize tumor-associated antigens (TAAs) through their T cell receptors (TCR). Cytotoxicity is triggered upon binding of antigen fragments presented by antigen presenting cells (APC) via MHC class I molecules. The other important players are the natural killer (NK) cells that recognize and exert cytotoxic activity through diverse activating and inhibiting receptors, which recognize specific ligands on the surface of target cells. Most tumor cells are destroyed in the elimination phase; however, some variants adapt to survive and may enter the next phase. The “equilibrium phase” is associated with regulatory pathways that maintain tumor cells in a state of immune-mediated dormancy [13], and that may last for the lifetime of an individual. The duration of this phase depends on the balance between the strength of the endogenous antitumor immunity and the immune tolerance of the tumor cells. This process leads to the emergence of tumor cell variants with reduced immunogenicity as a consequence of epigenetic alterations and genomic instability [14]. Under continuous selection pressure exerted by lymphocytes and cytokines, resistant tumor variants enter the “escape phase” in which they begin to grow progressively without the immunological constraints, establish an immunosuppressive TME, and give rise to clinically overt tumors [12]. These tumor variants are resistant to conventional therapies and are the main cause of mortality in cancer patients.

**Figure 1:**
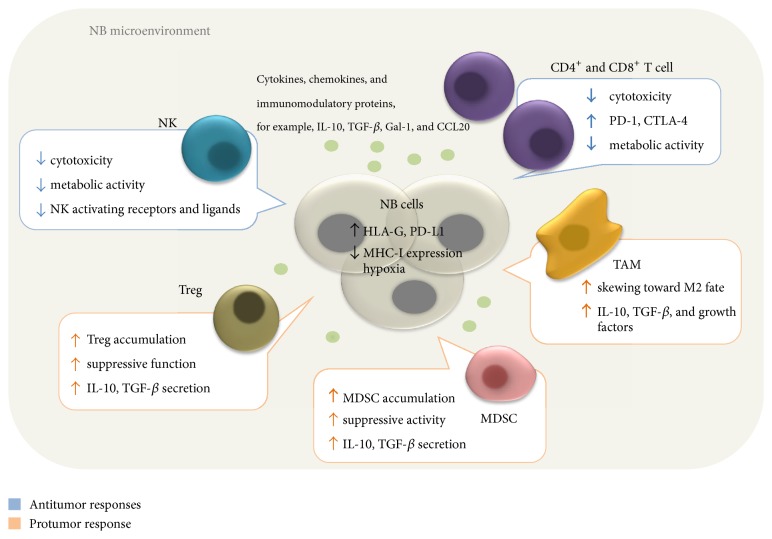
General model of the interactions between immune and cancer cells in the TME. NB cells play a central role in creating an immunosuppressive microenvironment. Hypoxia poses a metabolic challenge to infiltrating immune cells. NB cells also express membrane-bound and secreted immunosuppressive proteins such as IL-10 and TGF-*β*, which recruit Tregs, MDSCs, and TAMs and promote their suppressive activity, thus inhibiting the antitumor function of effector cells.

In this review, we provide an overview of the mechanisms of immune evasion present in the immunosuppressive microenvironment of NB (Figure 1), how it modulates the immune system and impacts negatively the antitumor immune response, and the development of therapeutic strategies to overcome tumor escape. It is also important to emphasize that further understanding and integration of fundamental knowledge of the tumor-host interaction are crucial to improving the potency of adoptive immunotherapy for children with NB.

## 2. Mechanisms of Immune Evasion

### 2.1. Infiltrating Immunosuppressive Cells in Neuroblastoma Microenvironment

Dysregulation of the balance between the effector and regulatory cell compartments is one of the main mechanisms for tumors to avoid immune eradication. Tumor-infiltrating lymphocytes (TILs) play a pivotal role in mediating antitumor immunity and in controlling cancer growth. During early neoplastic lesions, the infiltration of cytotoxic effector cells such as CD8^+^ T cells prevails; however, as cancer cells progressively grow, these cells are gradually outnumbered by immature cells of the innate immune system like tumor-associated macrophages (TAMs), type 2, and myeloid-derived suppressor cells (MDSCs) acquiring immunosuppressive phenotypes [15]. The presence of various infiltrating lymphocytes in primary tumors has been demonstrated in a number of studies and was associated with better clinical outcomes [16]. Although the role of these cells in NB patients remains to be fully elucidated, analyses of solid malignancies have allowed the identification of immune cells that have favorable and deleterious aspects on clinical prognosis [17]. In general, the presence of infiltrating CD8^+^ cytotoxic T cells, CD45RO^+^ memory T cells, CD4^+^ Th1 T cells, and NK cells served as a prognostic factor of favorable outcome in several cancers including breast, melanoma, ovarian, colorectal, and NB [17, 18]. On the other hand, a high level of immunosuppressive immune cells including TAMs, regulatory T cells (Tregs), and MDSCs may contribute to the generation of an immunosuppressive microenvironment, hindering effective anticancer immune responses, and thus may be associated with a poor clinical outcome.

Macrophages are the most abundant infiltrating stromal component within the TME. Macrophages are traditionally classified into two distinct populations, that is, M1 (or classically activated) and M2 (or alternatively activated) macrophages based on their functions and gene expression profiles. M1-polarized macrophages are induced by interferon *γ* (IFN*γ*) and lipopolysaccharides. This cell population produces immunostimulatory cytokines and exhibits tumor suppressive activities. On the other hand, M2-polarized macrophages activated by IL-4 dampen inflammatory responses and promote tumor cell immune evasion, invasion, and angiogenesis [19, 20]. Macrophages are generally known as TAMs when they present within tumors, and based on their functions, TAMs are closer to M2-polarized macrophages. TAMs express M2 markers (i.e., CD163, CD206) and produce immunosuppressive cytokines, for example, IL-4, IL-10, transforming growth factor-*β* (TGF-*β*), and secretory factors such as matrix metalloproteinases (MMPs), epithelial growth factor (EGF), and vascular endothelial growth factor (VEGF) that support tumor invasiveness and angiogenesis [21]. It has been reported that metastatic NB showed a higher degree of CD163-positive macrophage infiltration than locoregional tumors and the presence of high levels of these TAMs was associated with a prognostic signature [22]. Moreover, the expression of TAM associated genes such as CD33, CD16, IL6R, IL10, and FCGR3 enabled identification of a subgroup of patients with a poor outcome based upon tumor classification scores for predicting progression-free survival (PFS) [22]. A recent study showed that peripheral blood mononuclear cell- (PBMC-) derived macrophages stimulated with conditioned medium of a neuroblastoma cell line (NBCM) acquired M2 characteristics, which in turn stimulated an NB cell invasive phenotype [20, 23]. An immunohistochemical analysis of 41 NB cases revealed a significant association between CD163-positive macrophages and clinical features, supporting the findings of Asgharzadeh et al. (2012) [20, 22].

Tregs account for 5–10% of CD4^+^ T cells and play crucial roles in maintaining immune homeostasis, self-tolerance, and preventing autoimmunity. This T cell subset is generally characterized by expression of the transcription factor FOXP3, which is crucial for their suppressive activity, and interleukin-2 receptor alpha chain (CD25) [24]. Two main types of Treg are natural Treg (nTeg) that are thymus-derived and induced Treg (iTreg) arising from conversion of conventional CD4^+^ T cells exposed to tumor-derived factors. In cancer, Tregs comprise a “bad” subset and a “good” subset. Reports of various cancers have shown that an accumulation of Treg infiltrated into tumor tissues is often associated with poor prognosis [25] since Tregs contribute to cancer progression through their ability to suppress antitumor effector cell functions. However, it should be acknowledged that Treg infiltration can be associated with better prognosis in certain malignancies (such as colorectal, gastric, and triple negative breast cancer) via their ability to suppress cancer-mediated inflammation [24, 26]. The roles of Tregs in NB are still controversial. Only a few studies have investigated the association between Treg frequency and clinical outcomes in NB patients. An increased circulating Treg percentage has been found in NB patients as compared to healthy controls but did not correspond to prognostic factors [27, 28]. In another report, a lower frequency of both CD4^+^CD25^hi^CD127^−^ Treg cells and CD4^+^CD45R0^+^CD49b^+^LAG3^+^ type 1 regulatory (Tr1) cells subsets was observed in BM and peripheral blood (PB) samples from NB patients [29]. These discrepancies may be related to limited number of patients in the cohort studies, eligibility criteria, and the existence of different Treg subsets. Despite inconsistent data on the correlation between Treg frequency and clinical outcome, transient depletion of CD25^+^ and CD4^+^ using monoclonal antibodies can improve the efficacy of immunotherapy mediated by CD8^+^ T cells* in vivo* [30–32]. Further investigation of the intratumoral composition of Treg is needed to reveal the roles of this immune subset in NB patients.

Limited data is available on clinical significance of MDSCs in relation to NB. Myeloid progenitor cells originate in the bone marrow and migrate to different peripheral organs where they differentiate into granulocytes, macrophages, or dendritic cells (DCs) in healthy individuals [33]. In pathological conditions including cancers, chronic infectious diseases, and some autoimmune disorders, these cells differentiate into immature myeloid cells, namely, MDSCs [34]. They represent a heterogeneous population of cells with immunosuppressive properties. MDSCs possess immunosuppressive activities through several mechanisms: (i) inhibition of antigen-specific and nonspecific T cell activation via arginase- (ARG-) 1 and inducible nitric oxide synthase (iNOS); (ii) generation of reactive oxygen species (ROS); (iii) cysteine deprivation; (iv) induction of Tregs mediated by IL-10 and TGF-*β* [34, 35]. Accumulation of MDSCs was reported during tumor progression in NB mouse models [36] and promoted* in vivo* tumor growth through production of ROS, ARG-1, and TGF-*β* [37]. Treatment with low-dose aspirin was found to reduce tumor volume and display a reduced proportion of tumor-associated cells from the innate immune system, including MDSCs in the TH-MYCN transgenic mouse model for NB [36], suggesting that MDSCs may play roles in cancer-related inflammation to enhance NB progression.

### 2.2. Immune Evasion via Modulation of Antigen Presentation Machinery (APM)

Impaired antigen presentation is one of the most extensively studied mechanisms of immune evasion exploited by cancer cells. In general, antitumor activities strongly depend on the effectiveness of TAA presentation. Different TAAs have been identified from NB cell lines and primary tumors, including the ganglioside GD2, the glycoprotein CD56, melanoma antigen encoding gene- (MAGE-) A1, MAGE-A3/A6, NY-ESO-1, B melanoma antigen (BAGE), and G antigen (GAGE) [38–40]. TAAs originate from degradation of cellular proteins into short peptides by the proteasome in the cytosol. Peptides are then transferred into the lumen of the endoplasmic reticulum (ER) by the TAP transporter, loaded onto MHC class I (MHC-I) molecules composed of HLA class I heavy chain and *β*2-microglobulin (*β*2m), and subsequently transferred to the cell surface [41]. In addition, TAAs released from cancer cell death were processed and presented by DCs in order to prime and activate T effector cells, particularly CD8^+^ cytotoxic T lymphocytes. The activated tumor specific T cells then migrate and infiltrate into the tumor bed to recognize TAAs bound on MHC class I of cancer cells through their TCR, leading to T cell-mediated cytotoxicity [42]. Although the presence of TILs is often associated with better prognosis, it is worth mentioning that these TILs may become inactive at the tumor site in response to tumor-derived signals presenting in the TME.

Downregulation of MHC-I and molecules involving in TAAs processing and presentation may limit the effectiveness of antitumor immunity. Studies have found that NB displays low expression of MHC-I molecules and/or defects in some APM [43]. Mutations of the *β*2m gene, a component of the MHC-I molecule, can cause a complete absence of MHC-I expression [44]. Downregulation is also achieved by mutations in the TAP transporter and/or components of the immunoproteasome such as the latent membrane protein (LMP) 2 and LMP7. Note that expression of these components in NB cell lines could be restored by IFN*γ* treatment [41, 43].

NK cells can interact with MHC-I molecules through their killer cell immunoglobulin-like receptors (KIRs), which suppress their cytotoxic activity. This lymphocyte alters KIR expression to maintain the balance between defense and self-tolerance. Downregulation of HLA class I present on transformed cells leads to an absence of the inhibition signal, which in turn sensitizes those tumor subpopulations to NK cell-mediated cytotoxicity [41]. However, tumor cells operate another mechanism to modulate NK cell activity. NKG2D and DNAM-1 are NK cell activating receptors that play important roles in NK cell-mediated recognition and killing [41, 45]. Downregulation or shedding of NK cell activating ligands can therefore reduce cancer cell killing mediated by NK cells. MYCN amplification, a well-established predictor of poor prognosis in NB, may serve as a negative regulator of NKG2D ligands, that is, MIC-A, MIC-B, ULBP-1, ULBP-2, and ULBP-3, and DNAM-1 ligand, for example, PVR [45, 46], thus supporting the role of MYCN as an immunosuppressive oncogene in high-risk NB patients.

HLA-G has been reported to play antitumor role in cancer [47–50]. HLA-G is the best characterized nonclassical HLA class Ib, a subfamily of MHC class I molecules, which includes HLA-G, HLA-E, HLA-F, and HLA-H molecules. HLA-G has 7 protein isoforms (HLA-G 1–7) derived from alternative splicing of the primary transcript generating both membrane-bound and soluble proteins [48]. This protein can interact with inhibitory receptors on a wide range of immune effector cells, including T and B lymphocytes, NK cells, DCs, granulocytes, monocytes, and macrophages [48, 49]. In patients with NB, higher soluble HLA-G (sHLA-G) concentration in plasma may be associated with poorer outcome. Morandi et al. reported that sHLA-G was released by both NB cells and monocytes upon stimulation by conditioned medium from NB cell lines [49]. The same group of investigators also demonstrated that sHLA-G concentration in bone marrow plasma samples was higher in NB patients with metastatic disease than patients with localized NB [50]. The sHLA-G isoforms can inhibit NK and T cell functions by inducing apoptosis as well as inhibiting B cell proliferation [49], pointing to possible correlation between HLA-G concentration and disease progression.

### 2.3. Secreted Immunosuppressive Factors

The cellular components of the tumor are composed of both cancer cells and host components. A variety of soluble molecules are secreted in the TME from both cancerous and noncancerous cells to stimulate cancer progression including proliferation, chemoresistance, antiapoptosis, migration, and invasion. Among these, TGF-*β*, IL-10, and secreted galectin-1 have been detected and found to mediate immunosuppression in the NB microenvironment [51–59].

TGF-*β* is a multifunctional immunosuppressive cytokine that inhibits T, B, and NK cell function and promotes the function of Tregs [48]. CD4^+^ T cells can differentiate into iTregs in the presence of TGF-*β*. In addition, TGF-*β* acts directly on NB cells to regulate cell proliferation and differentiation [53]. The molecular mechanism mediated by TGF-*β* on CD8^+^ T cells involves inhibition of production of perforin, granzymes A and B, the proapoptotic cytokines Fas-ligand, and IFN*γ* [54]. TGF-*β* also dampens T cell activation by impairing DC function.

IL-10, also known as human cytokine synthesis inhibitory factor (CSIF), is secreted by a wide variety of cells in an immune response. A gene expression study reported higher IL-10 mRNA expression in metastatic NB patients than those in controls with no apparent association with clinical outcome [27]. Similar to TGF-*β*, IL-10 inhibits the function of DCs and macrophages and thus indirectly prevents antigen-specific CD4^+^ T cell activation and also promotes M2 polarization.

Galectin-1 (Gal-1) belongs to a family of carbohydrate-binding proteins with a wide range of biological activities. This protein plays multiple roles in tumor progression including cellular adhesion, cell motility, angiogenesis, chemoresistance, and most importantly immunomodulatory effects [55]. Intracellular Gal-1 is involved in signaling pathways, whereas extracellular Gal-1 protein interacts with cell surface glycoproteins, forming multivalent complexes on the cell surface termed “lattices.” Several lines of evidence show increased extracellular Gal-1 in many types of cancer and its overexpression is associated with poor prognosis [55]. Gal-1 is secreted not only by cancer cells but also by stromal cells surrounding tumor including monocytes, macrophages, T lymphocytes, and fibroblasts [56]. Gal-1 secreted by tumor cells triggers T cell apoptosis, inhibits DC maturation, and contributes to polarization of macrophages from M1 to M2 [57, 58]. Knockdown of Gal-1 in the high Gal-1 expressing NXS2 mouse NB cells resulted in increased levels of IFN*γ* and significantly higher frequency of infiltrating T cells. Supernatants of wild type NXS2 cells also suppressed DC maturation and induced T cell apoptosis [58]. Differences in Gal-1 functions based on the producing cells have been reported. Gal-1 deficiency in CD4^+^ T cells was shown to impair T cell migration to the tumor site, whereas tumor-derived Gal-1 was shown to promote metastases accompanied by reduced tumor infiltration by immune cells [59].

### 2.4. Tumor Cell Metabolism

Distinctive features of tumor cell metabolism can promote an immunosuppressive microenvironment and immune evasion. As carcinogenesis begins, rapid tumor growth and aberrant vasculature formation lead to an inadequate oxygen and nutrient supply in the TME. Since hypoxia is a common feature of solid tumors, the involvement of hypoxia in cancer metastasis has been relatively well studied. In this hostile microenvironment, cancer cells undergo metabolic reprogramming by switching from mitochondrial oxidative phosphorylation to aerobic glycolysis, termed “the Warburg Effect.” Conversion of pyruvate to lactate by lactate dehydrogenase (LDH) results in local acidity. Tumor hypoxia greatly influences most of the cancer “hallmarks,” that is, cell proliferation, differentiation, invasion, metabolism, and chemoresistance [60]. The cellular response during hypoxia is generally mediated by the hypoxia-inducible factor (HIF) family of transcription factors, which regulate expression of various target genes. Hypoxia is well established to confer a more aggressive phenotype and may act as a marker of poor prognosis in NB [61]. However, the effect of a hypoxic tumor on immune evasion in NB is still unclear. Low oxygen availability accompanied by an acidic pH has profound effects on both innate and adaptive immune cells. An insufficient supply of oxygen can affect T cell differentiation and function, possibly skewing T cell fate toward a T helper (Th) 17/Treg phenotype and impairing NK cell cytotoxic properties [62]. Hypoxic stress also promotes the acquisition of progressive immune escape via the recruitment of MDSCs, TAMs, and Tregs [63]. HIF-1*α* significantly increased the expression of programmed cell death ligand 1 (PD-L1) and the secretion of IL-6, IL-10, and TGF*β*1 in MDSCs in tumor bearing mice [62]. The binding of the PD-1/PD-L1 system reduces the effector functions of T and NK cells. Furthermore, hypoxia induces M2-like polarization of TAMs and upregulation of arginase I, IL-10, and TGF*β* [63]. NB intratumoral hypoxia triggers induction of CCL20 expression in TAMs. The same chemokine is utilized by NKTs to migrate to the tumor site and CCL20 expression was believed to trap NKT cells in the hypoxic tissues and disable their function [64].

Arginine metabolism has emerged as a key regulator of immune responses in cancer biology. Myeloid cells are the main players that use arginine metabolism via NOS and arginase to mediate diverse immunological consequences. Arginase 1 mediated arginine depletion is one of the first mechanisms of T cell suppression described in MDSCs where low level of arginine results in inhibitions of T cell receptor expression and antigen-specific T cell responses [65]. In addition to cells of the myeloid lineage, NB cells were found to upregulate arginase 2, which catalyzes the conversion of arginine into ornithine and urea [66]. Lower arginine concentration in TME may inactivate T and myeloid cells, hence decreasing tumor infiltration of these immune cells [66].

## 3. Overcoming the Immunosuppressive Tumor Microenvironment and Future Prospects

**Figure 2:**
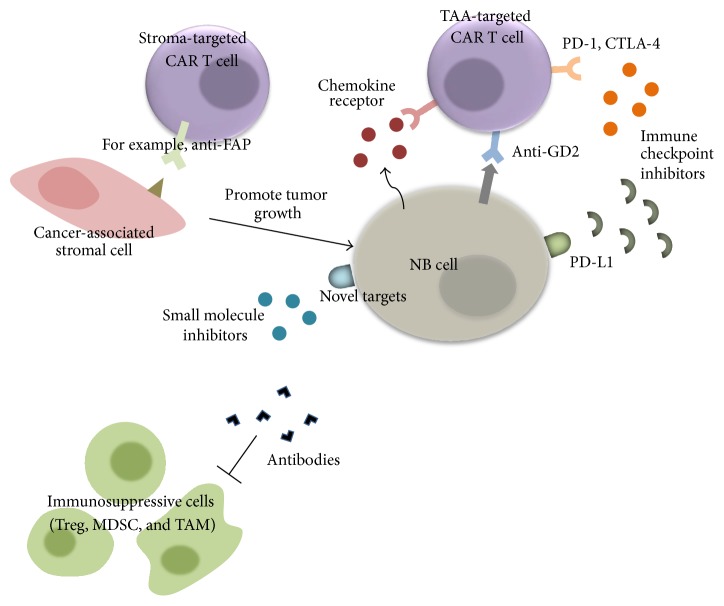
Therapeutic strategies to overcome the immunosuppressive TME. Combinatorial therapeutic approaches where CAR T cells directed against TAA are administered simultaneously with stromal targeted therapy represent the future of NB treatment. CAR T cells can be genetically modified to express various kinds of receptors including a chemokine receptor, a dominant negative receptor, or receptor targeted TME components. These can be provided in combination with other types of targeted therapy such as antibodies, small molecule inhibitors, and/or immune checkpoint inhibitors.

In the past decades, tremendous effort has been put into immunotherapeutic approaches to cancer treatment. Overwhelming evidence supporting the critical role of the immune system in tumor eradication combined with the modern molecular tools prompts us to create genetically engineered T lymphocytes directed against specific antigens, namely, CAR T cells. The use of CAR enables T cells to recognize TAAs in an MHC-independent manner, hence overcoming defects in antigen processing and presentation mediated by tumor cells, one of the inhibitory mechanisms for initial tumor escape. CAR T cells have demonstrated clinical efficacy in a number of hematological malignancies [67], while the same approach for solid tumors is less developed. CAR T cells have been tested in a few clinical trials for NB patients with suboptimal outcomes [68]. Indeed, the immunosuppressive TME constitutes a major obstacle to adoptive T cell therapy for NB. As we are now moving toward an era of personalized medicine, the use of combinatorial therapeutic platforms tends to be the superior choice for cancer treatment, to overcome tumor heterogeneity. Research has now focused on combining modality regimens to augment and prolong antitumor efficacy of adoptive transfer therapy while concomitantly targeting tumor-associated stroma to overcome tumor escape mechanisms (Figure 2).

CAR T cell trafficking and accumulation at the tumor tissue are the prerequisites for optimal antitumor response. Many approaches have been taken to circumvent poor cell trafficking in the TME including the regional delivery of CAR T cells and transgenic expression of chemokine receptors on these effector cells [69]. Adoptive transfer of NKT cells modified to express IL-15 showed protection of NKT cells from the inhibitory effect of hypoxia and enhance antitumor activity in a metastatic NB model [64]. Transgenic expression of CCR2b on CAR T cells significantly promotes both* in vitro* and* in vivo* chemotaxis in response to CCL2 secreted by NB cells and improved migration ability is also associated with greater antitumor efficacy [70].

TILs present within the TME undoubtedly have a great impact on tumor prognosis and response to therapy. Although tumor infiltration by T, NK, and NKT cells is associated with improved prognosis, disruption of effector function at the tumor site poses a major challenge in cancer treatment. Preclinical and clinical data revealed that CAR T cells progressively lose their function following infusion, termed T cell exhaustion [71]. The phenomenon occurs when T lymphocytes lose their effector function and remain hyporesponsive as a consequence of continuous TCR stimulation from persistent TAA [71]. Exhausted T cells are characterized by expression of immune checkpoints such as programmed cell death protein 1 (PD-1), cytotoxic T lymphocyte-associated antigen 4 (CTLA-4), and lymphocyte activation gene 3 (LAG-3).

PD-1 and CTLA-4 are receptors in the CD28 ligand-receptor family providing inhibitory signals to T lymphocytes. Overexpression of these two receptors in the TME contributes to inhibition of antitumoral immune response [62]. PD-1 binds to two ligands known as PD-L1 and PD-L2. The receptor is expressed by activated T and Treg cells and also on other immune cells such as activated B and NK cells. The main function of PD-1 mediated cellular response is the control of T cell activation and the maintenance of immune tolerance to self-antigens. The PD-1/PD-L1 pathway can induce T cell apoptosis or dysfunction [71]. Interaction of PD-1 with its ligand confers a different effect in Tregs, where the binding promotes Treg cell proliferation and enhances their immunosuppressive function [48]. PD-L1 was found to be expressed in several cancer types including ovarian, breast, cervical, colorectal, pancreatic, and gastric cancer, melanoma, and glioblastoma in response to inflammation, whereas PD-L2 is expressed on DC, macrophages, mast cells, and B cells [72]. Given that, immune checkpoint inhibitors are being developed for clinical use. They can be classified into two categories: anti-PD1 and anti-PD-L1 antibodies. The antibodies have been approved by US Food and Drug Administration (FDA) for treatment of solid tumors [73]. Less is known about expression of PDL1 in NB. To date, inconsistent data has been published regarding PD-L1 expression from NB samples. Aoki et al. (2016) did not find PD-L1 expression in any of 18 samples tested [74]. In contrast, Chowdhury et al. (2015) reported PD-L1 expression in 72% of high-risk NB (31/43) [75]. PD-1 checkpoint blockage in combination with CAR T cells has been demonstrated to be beneficial in other types of solid tumors such as colon, renal, lung, and breast cancer [48, 76]. CTLA4 competes with CD28 for the binding of CD80 and CD86 expressed by antigen presenting cells. CTLA4 is expressed in activated T cells and constitutively expressed in Tregs. Similar to PD-1, the function of CTLA4 is to prevent overactivation of the immune response. Blockage of CTLA4 increases antitumor response and attenuates tumor progression [77]. Success in a clinical trial in metastatic melanoma patients led to FDA approval of Ipilimumab [78]. The study reported an improvement of overall survival rate by approximately 4 months (10 versus 6.4 months) when Ipilimumab was added to the regimen. Two humanized anti-CTLA-4 antibodies, Ipilimumab and Tremelimumab, have been approved as therapeutic options for the treatment of cancer.

Unlike cancerous cells, the stromal components are genetically stable; therefore risk of treatment resistance or emergence of new genetic variants can be minimized [79]. Small molecule inhibitor or anticytokine therapy coadministered with adoptive transfer is a promising approach for the treatment of solid tumors. To date, various small molecule drugs are being used to target the tumor stromal components including the lymphatic vessels, vasculature, TAMs, and Cancer-Associated Fibroblasts (CAFs). A search for novel therapeutic targets is also actively ongoing, which can engender more effective and more personalized interventions. Potential targets are generally tumor-promoting factors present in the TME such as IL-6, TGF-*β*, Gal-1, and Gal-3 [57, 80, 81]. Moreover, depletion of immunosuppressive cell populations, Tregs, MDSCs, and TAMs, by specific antibodies has been shown to confer some benefits in immunotherapy for breast cancer, leukemia, myeloma, fibrosarcoma, colon adenocarcinoma, glioma, and lung cancer [79, 82].

More recently, various types of CAR are continuously being developed to battle against immune evasion mechanisms and to further enhance antitumor efficacy of adoptive T cell therapy. One of the recent approaches has focused on developing CAR T cells specific for stromal cells. For example, fibroblast activation protein (FAP) is a transmembrane serine protease expressed in the cancer-associated stromal cells (CASCs) that emerged as a therapeutic target. Inhibition of FAP resulted in tumor growth inhibition [83]. A similar effect was reported from CAR T cell targeted FAP; a treatment with the FAP-CAR T cells resulted in ~80% depletion of FAP^+^ cells, which was associated with a significant inhibition of tumor growth (35–50%) in mesothelioma and lung cancer mouse models [84]. Another attractive target is vascular endothelial growth factor receptor (anti-VEGFR2). VEGF and its receptor, VEGFR-2, have the immune suppressive effect on various immune cells. VEGF has been reported to inhibit maturation of DC, disrupt the infiltration and function of T cells, and induce Treg function [85]. Disruption of VEGF/VEGFR-2 signaling by simultaneous transfer of CAR T cells expressing anti-VEGFR-2 and T cells specific for gp100 (PMEL), TRP-1 (TYRP1), or TRP-2 (DCT) significantly eradicated B16 melanoma tumors in mice [86]. Overall, studies have shown that combining tumor and stroma reactive CAR T cells exhibited synergistic antitumor activity compared to treatment with either cell type alone [86, 87]. Upon entering the TME, T cells inevitably face immunosuppressive molecules such as TGF-*β* and IL-10. The development of T cells armed with a dominant negative TGF-beta receptor (a human TGF-*β* receptor with a truncated endodomain) has conferred tumor-derived TGF-*β* resistance to antigen-specific cytotoxic T lymphocytes (CTLs) [88]. Another strategy to counteract the hostile TME milieu is to generate a chimeric cytokine receptor that converts an immunosuppressive signal into a positive signal. In one study, the exodomain of the receptor for the antiproliferative cytokine IL-4 was genetically engineered to fuse to the endodomain of the Th1 proliferative cytokine, IL-7. These transgenic T cells have demonstrated improved proliferation and survival both* in vitro* and* in vivo* [89].

## 4. Conclusion

High-risk NB is one of the most difficult childhood cancers to treat. The concept of immune surveillance suggests a positive role for immune cells in controlling cancer progression. Based on that observation, research has been focused on harnessing the immune system to fight against cancers. A paradigm shift in cancer treatment has been achieved through the development of CAR T cells and identification of TAAs. However, challenges have been faced when translating CAR T cells to clinical trials, particularly for solid tumors. CAR T cells will be susceptible to becoming hyporesponsive upon entering the suppressive TME. Consequently, adoptive immunotherapy for NB has been disappointing. Several immune escape mechanisms employed by NB cells have been discussed, that is, recruitment of immunosuppressive cell populations, perturbations of APM components, and secretion or expression of immunosuppressive factors and metabolic alternations of cancer cells. These factors play an important role in creating an immunosuppressive cellular network, hence disrupting effective antitumor immunity. More advanced approaches to enhancing CAR T cell function and survival* in vivo* are being explored. These include luring T cells with chemokine receptors, targeting immune checkpoints, and the TME-targeting therapies. More importantly, a deeper understanding of the key immune players and the regulatory pathways involved in the complexity and dynamic interaction among tumor cells and the immune system is crucial for the identification of prognosis factors and advancement of therapeutic strategies to boost the immune system against cancers. It is believed that the future of immunotherapy for NB will lean toward combination therapy where cancer cell-directed agents are cotransferred with a therapeutic regimen targeting the TME, to provide long-lasting and effective antitumor immunity.
